# Identifying Influence Agents That Promote Physical Activity Through the Simulation of Social Network Interventions: Agent-Based Modeling Study

**DOI:** 10.2196/12914

**Published:** 2019-08-05

**Authors:** Thabo J van Woudenberg, Bojan Simoski, Eric Fernandes de Mello Araújo, Kirsten E Bevelander, William J Burk, Crystal R Smit, Laura Buijs, Michel Klein, Moniek Buijzen

**Affiliations:** 1 Behavioural Science Institute Radboud University Nijmegen Netherlands; 2 Social AI Group Vrije Universiteit Amsterdam Netherlands; 3 Radboud Institute for Health Sciences Radboud University and Medical Centre Nijmegen Netherlands

**Keywords:** physical activity, adolescent health, peer group, computer simulation, social network analysis

## Abstract

**Background:**

Social network interventions targeted at children and adolescents can have a substantial effect on their health behaviors, including physical activity. However, designing successful social network interventions is a considerable research challenge. In this study, we rely on social network analysis and agent-based simulations to better understand and capitalize on the complex interplay of social networks and health behaviors. More specifically, we investigate criteria for selecting influence agents that can be expected to produce the most successful social network health interventions.

**Objective:**

The aim of this study was to test which selection criterion to determine influence agents in a social network intervention resulted in the biggest increase in physical activity in the social network. To test the differences among the selection criteria, a computational model was used to simulate different social network interventions and observe the intervention’s effect on the physical activity of primary and secondary school children within their school classes. As a next step, this study relied on the outcomes of the simulated interventions to investigate whether social network interventions are more effective in some classes than others based on network characteristics.

**Methods:**

We used a previously validated agent-based model to understand how physical activity spreads in social networks and who was influencing the spread of behavior. From the observed data of 460 participants collected in 26 school classes, we simulated multiple social network interventions with different selection criteria for the influence agents (ie, in-degree centrality, betweenness centrality, closeness centrality, and random influence agents) and a control condition (ie, no intervention). Subsequently, we investigated whether the detected variation of an intervention’s success within school classes could be explained by structural characteristics of the social networks (ie, network density and network centralization).

**Results:**

The 1-year simulations showed that social network interventions were more effective compared with the control condition (beta=.30; t100=3.23; *P*=.001). In addition, the social network interventions that used a measure of centrality to select influence agents outperformed the random influence agent intervention (beta=.46; t100=3.86; *P*<.001). Also, the closeness centrality condition outperformed the betweenness centrality condition (beta=.59; t100=2.02; *P*=.046). The anticipated interaction effects of the network characteristics were not observed.

**Conclusions:**

Social network intervention can be considered as a viable and promising intervention method to promote physical activity. We demonstrated the usefulness of applying social network analysis and agent-based modeling as part of the social network interventions’ design process. We emphasize the importance of selecting the most successful influence agents and provide a better understanding of the role of network characteristics on the effectiveness of social network interventions.

## Introduction

### Background

There has been an increasing interest in the use of *social network interventions* to promote health behaviors. Social network interventions are based on the diffusion of innovations theory [1] and capitalize on interpersonal influence to promote and catalyze desired behavioral changes [2]. A few studies have used social network interventions to promote health behaviors in school settings [3]. For example, the A Stop Smoking In Schools Trial study trained influence agents to encourage peers not to smoke in secondary schools [4]. Other studies have trained influence agents to stimulate peers to increase health behaviors, such as drinking more water [5] or being more physically active [6,7].

One of the most important assumptions of social network interventions is that some peers act as role models and can be important determinants of the behavior of the group [8]. Involving these important peers in the intervention can prove beneficial, as they can be used as an example for the rest of the social network; they can help ensure that the intervention message spreads among the individuals in the social network. In such an intervention, the health behavior is disseminated among the classmates through their network ties [1] and will lead to less resistance. Therefore, in most social network interventions, a subset of participants is selected as *influence agents* to initiate the diffusion of an idea or behavior. The influence agents can volunteer or be appointed by researchers, but many social network interventions rely on peer nominations within a social network to determine the influence agents [2]. Participants nominate peers on a number of questions (eg, “Who are your friends?”). On the basis of these nominations, 10% to 17.5% of individuals are approached to become influence agents [2]. The influence agents are trained to adopt and spread a new or improved health behavior or informally diffuse the intervention messages within their social network. However, it is not yet clear which individuals in a network make the most effective influence agents. In other words, what is the best selection criterion to determine influence agents?

An ideal solution to this question would be to run a large-scale field experiment with different criteria for selecting the influence agents. However, this would be a costly undertaking, which is probably the reason why this question has remained unanswered. Fortunately, advancements in computer science have enabled us to simulate hypothetical social network interventions by using computational models [9,10]. This contemporary approach is a big step forward in the intervention studies’ design process. Computational models can be a promising method to understand the complex interplay between social influences and other factors that are driving certain health behaviors [11]. For example, researchers can collect baseline data, simulate a wide range of interventions, and opt for the intervention strategy with the biggest changes in behavior or the one that is most cost-effective. In addition, computational models could be used in consultation with key stakeholders to determine priorities, create expectations about the interventions, and tackle issues regarding implementation early on. Finally, simulations enable researchers to formulate data-driven hypotheses that can be tested in vivo. Therefore, computational models are a valuable addition to the toolbox of researchers and practitioners who aim to change behaviors.

Agent-based models (ABMs) are used to model interactions among individuals within a social network and, therefore, fit the theoretical underlying mechanisms of social network interventions. The behavior of an influence agent has an effect on the individuals with whom the influence agent shares a connection. To develop effective social network interventions, it is essential to understand how behavior spreads in a social network and what affects the spread of the desired behavior. ABMs are a helpful tool for this, as they enable researchers to experiment in simulated environments. In previous research, ABMs were used to ascertain effective ways of identifying important influencers [10,12-14]. In addition, ABM simulations have been increasingly explored as an alternative approach for addressing health research questions. Furthermore, previous studies have shown that ABMs can be used to model physical activity behavior [15-17] or obesity [18,19] in a social network.

The aim of this study was to test which selection criterion to determine influence agents in a social network intervention resulted in the biggest increase in physical activity in the social network. An ABM was used to test different selection criteria for influence agents by simulating social network interventions and observing the intervention’s effect on the physical activity within school classes. In this study, we relied on the methods and model specifications of our previous study [20] to build the social networks and implement the computational model. Drawing on a previously validated model developed by Beheshti [12] and Giabbanelli et al [21], the computational model employed in this study was applied to the observed data of primary and secondary school children collected in the *MyMovez* project [22]. The model considered 2 factors as determinants for an individual’s behavioral change: the class’s social influence and the individual’s social environment (for more information see [20]). In the model, the behavior of influence agents has an effect on those with whom he or she shares a relationship: the effect of the influence agents spreads from connection to connection. This is referred to as *social network influence*. In addition, the ABM used in this study considered the influence of the physical environment as a potentially important factor for promoting health behavior.

To further investigate the applicability of ABMs for social network interventions, this study examined whether the simulated effectiveness of social network interventions was dependent on several network characteristics. We built upon Valente's idea that the interventionist should not only use the networks as an intervention instrument but also learn from the available social network information to create better, meaningful interventions [20]. In addition, Giabbinelli et al [21] concluded that there are microlevel network structures to be investigated, which are involved in making the agents more resilient to change. Other studies also state that interventions might be less effective if they neglect the impact of social networks [23]. Therefore, we investigated if characteristics of social networks (classes) could affect the effectiveness of network-based health interventions.

The analysis presented in this study is based on the *MyMovez* dataset of 26 school classes. This provided a rare opportunity to simulate social network health interventions in school classes based on a comprehensive real-world dataset with real social networks and physical activity data. Multiple sociometric nominations submitted by the participants were used to define weighted relationships among the class peers and thereby build the social networks. Physical activity and social environment data were used to define the state of each agent per day.

In this study, we identified 2 sets of hypotheses. First, we compared the outcomes of different conditions to determine the selection criteria for most effective influence agents in social network interventions. The effectiveness of the interventions was measured by the difference in physical activity between the baseline and after 1 year of simulation. Second, this study investigated whether different network characteristics (ie, density and centralization) of the classes could affect the effectiveness of social network interventions.

This study is a product of collaborative research between social and computer scientists, with the motivation to translate the findings into applicable advice for preparing network-based interventions. The social and computer science research communities examine social networks and network-based health interventions from fairly contrasting angles. Significant improvements could ensue with respect to the way social network health interventions are designed and implemented owing to strong collaborations between social and computer science research communities.

### Selecting Influence Agents

To assess the predictive validity of the computational model, the simulated interventions were compared with the no intervention condition. On the basis of social network theory and the overall positive outcomes of previous social network interventions [4-6,24], we expected a bigger increase in physical activity in the intervention conditions than in the no intervention condition.

Subsequently, we looked at selecting strategically placed influence agents, compared with having a random allocation of influence agents. Scholars have elaborated on different roles and positions of individuals within social networks (for an overview see [25]). Influence agents are often defined as individuals who are most *central* in the network [3]. This means that those individuals hold a prominent place in the network. Centrality is a measure of an individual's position relative to their social network, but there are a handful of definitions and algorithms used to define and measure centrality [3,25]. These definitions all assume that in one way or another, being central in the social network means that an individual is more influential. Therefore, we assumed that having central individuals as influence agents (regardless of the used definition) should increase the effectiveness of a social network intervention. Thus, we defined our first hypothesis as follows:

H2: The increase in physical activity will be higher in the simulated social network interventions based on centrality than in the simulated random influence agent intervention.

As Freeman [25] discussed, there is no consensus on a common definition of centrality or how it should be measured. There are 3 widely used definitions of centrality: in-degree centrality, betweenness centrality, and closeness centrality [3,26].

#### In-Degree Centrality

The most often used centrality measure in the social network interventions literature is *in-degree centrality*. In-degree centrality is based on the number of peer nominations an individual receives (notably, this is referred to as out-degree centrality when researchers use influence models instead of nomination models). The more the incoming peer nominations, the higher the in-degree centrality. So, individuals with high in-degree centrality can be seen as an important channel of information [25]. In school settings, most often the in-degree central influence agents are the most popular children or adolescents and are clustered together in the network. Therefore, the intervention could affect that small cluster of individuals and not reach the important subgroups or peripheral nodes in the network (who might benefit the most from the intervention). In addition, popular peers may be reluctant to change their behavior or perform the role of an influence agent [27]. The popular peers have a large contribution to the social norms within the network, and deviating from the established social norm could have a negative effect on their social status. Therefore, Borgatti [26,28] argues that 2 other types of centrality are likely to be more important for the promotion of health behaviors: betweenness centrality and closeness centrality.

#### Betweenness Centrality

Betweenness centrality focuses on the role of influence agents as a gatekeeper of information within social networks. These influence agents are important for linking different individuals, groups, or subgroups together and are referred to as being a *bridge*. More specifically, betweenness centrality is based on the frequency with which an individual is a link in the shortest path between 2 other peers. This means that this individual controls the flow of information among other peers in the network. Such an individual can influence the network by withholding or distorting information in the diffusion. If the betweenness central agents are not selected to disseminate the intervention message, entire subgroups could be withheld from the intervention [25]. In particular, Borgatti argues that betweenness central agents should be used when the goal is to disrupt the network’s ability to spread unhealthy behavior [28]. By removing these individuals from the social network, the residual network has the least possible cohesion and, therefore, will decrease the spread of negative behaviors in the network the most. In practice, it is not feasible to remove those individuals from a network but increase their physical activity to prevent a potential negative behavior (low physical activity) from spreading in the social network.

#### Closeness Centrality

Closeness centrality focuses on the reach of the influence agents within networks and dissemination speed of the intervention in the network. Closeness centrality represents the distance between the individuals and all other peers in a network. More specifically, closeness central individuals have on average the shortest path to all other peers in a network. This means that the intervention will reach the entire network in the least amount of links, and it makes the intervention message most efficient. Therefore, Borgatti argues that closeness central influence agents should be used when the goal is to promote positive health behaviors [28]. The positive intervention message will reach all members of the social network in the most efficient way and will not exclude clusters of or subgroups from the intervention message. This approach fits within the notion that to reduce weight, it is more effective to promote a healthy behavior (eg, physical activity) than to discourage negative behaviors (eg, watching television) [29]. Because the simulated social network interventions entail the promotion of physical activity (ie, a positive behavior), we defined our second hypothesis as follows:

H2: The increase of physical activity will be higher for simulated social network intervention based on closeness centrality than in simulated social network intervention based on in-degree and betweenness centrality.

### Network Characteristics

Next to the measurement of network properties at the individual level, social network analysis can also be used to describe network properties at the group level. It is important to understand group-level network information to create better and more meaningful interventions [30,31]. Because all classes are unique in their network properties, social network interventions should keep the structure of the network in mind. *Density* and *centralization* are 2 of the most important network characteristics that could influence the effectiveness of a social network intervention [32].

#### Density

**Figure 1:**
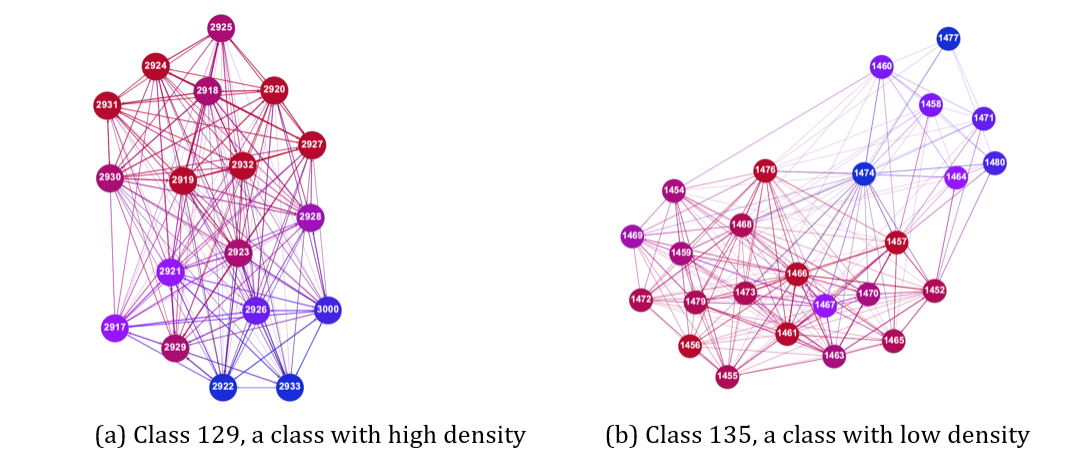
Examples of density in social networks.

The density of a social network is a measure of the cohesion in a network and can be defined as a ratio between the number of ties between participants and the number of all possible ties in a network. This means that dense classes have a relatively high number of connections among the individuals and thus have a high degree of cohesion. Figure 1 provides examples of social networks of 2 classes. The node color refers to the individual’s in-degree centrality. Red means a higher in-degree and blue means a low in-degree centrality. Ties between nodes are weighted based on 6 nomination questions, and participants could nominate an unlimited number of peers. The left network in Figure 1 is a classroom with high density as 90% of all possible ties are connected. The right network scores low on centrality, as only 46% of all possible ties are connected.

Networks with high density imply more peer interactions, therefore maximizing the opportunities for spreading an intervention within a social network [33]. We expected that this would also apply to social network interventions that promote physical activity; therefore, we defined our third hypothesis as follows:

H3: The effect of the simulated social network interventions will be higher in classes with high density than in classes with low density.

#### Centralization

*Centralization* of a network describes the distribution of the individual *centrality* measures of the participants in a network. In contrast to centrality, centralization is a network-level measure. Freeman describes centralization as the skewness of the distribution of nominations in a social network [25]. Centralization defines the extent to which interactions are concentrated in a small number of individuals rather than distributed equally among all peers [32]. This means that in highly centralized networks, there is a pronounced subgroup of central individuals. Network centralization can be calculated for all the centrality measures (ie, in-degree, betweenness, and closeness centrality).

**Figure 2:**
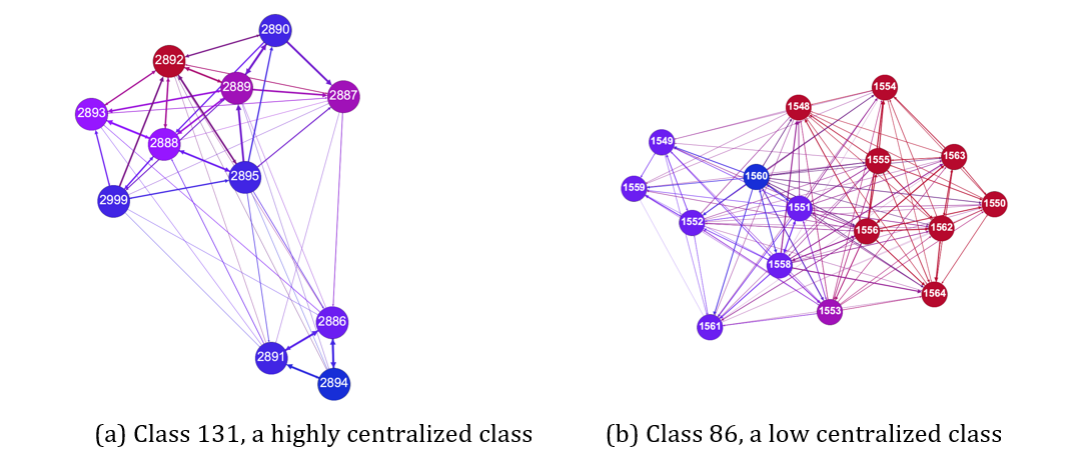
Examples of in-degree centralization in social networks.

Figure 2 is an example of in-degree centralization in 2 of the classes. The node color is proportional to the individual’s in-degree centrality. Red means a higher in-degree and blue means lower in-degree centrality. The left network in Figure 2 is an example of a class with high in-degree centralization. As can be visually observed, there is 1 individual (ID 2892) in the left social network who received proportionally more nominations than the rest of the class. Therefore, this class has a high score in in-degree centralization, and ID 2892 should be an effective influence agent in this class. In contrast, the network on the right has low in-degree centralization because it has a large subgroup of individuals who are high in in-degree centrality. The same principle applies to betweenness centralization and closeness centralization.

Previous research has shown the moderating role of centralization in the relationship between friendship networks and bullying in children [34]. More specifically, the centralization of the class predicted whether popularity related to aggressive behavior in boys. However, it has not been studied before whether social network interventions have more effect in centralized classes than in classes in which the nominations are spread evenly. We argue that classes with high centralization lend themselves better for social network interventions because the influence agents are more pronounced and, therefore, easier to detect by the researcher. These influence agents in centralized classes will have relatively more influence on the network than the influence agents in noncentralized classes. Therefore, we defined our last 3 hypotheses as follows:

H4a: The effectiveness of the simulated social network interventions based on in-degree centrality will be greater in classes with high in-degree centralization than in classes with low in-degree centralization.

H4b: The effectiveness of the simulated social network interventions based on betweenness centrality will be greater in classes with high betweenness centralization than in classes with low betweenness centralization.

H4c: The effectiveness of the simulated social network interventions based on closeness centrality will be greater in classes with high closeness centralization than in classes with low closeness centralization.

## Methods

### Participants and Procedure

The study used data from *MyMovez* project [22], a large-scale cross-sequential cohort study among children and adolescents (aged 8-12 and 12-15 years) from 21 primary and secondary schools [22]. In the project, participants received a smartphone with a research app on which they received daily questionnaires and a wrist-worn accelerometer (Fitbit Flex). This accelerometer has been shown to be a reliable measure of physical activity [35,36]. For this study, the first 4 waves of the *MyMovez* project were used: February to March 2016 (Wave 1), April to May 2016 (Wave 2), June to July 2016 (Wave 3), and February to March 2017 (Wave 4). To ensure that the influence agents are identified from a representative sample within each classroom, only classes with more than 60% of students participating were included. This resulted in 26 classes, with 460 participants (mean age 10.81 years, SD 1.28; male=51.5% [237/460]) in total.

### Measures

#### Physical Activity

In each wave, participants wore the accelerometer on their nondominant hand for 7 consecutive days. The first and the last day were excluded because these were partial days (handing out and giving back the accelerometer), resulting in 5 complete days of data. In addition, days that did not add up to 1440 min (24 hours) and days with less than 1000 steps were excluded because these were partial days of data (eg, caused by empty battery or nonwear time).

The average physical activity per wave was calculated by taking the average steps per day of at least 3 days of valid data. If participants had less than 3 days of valid data per wave, daily step count was imputed with the same strategy as in the study by van Woudenberg et al [7], by using single multilevel (predictive mean matching) imputation [37]. Missing data were imputed based on other physical activity data of the same participant, day of the week, measurement period, sex, and age. On average, participants accumulated 10,505 steps per day (SD 5.730).

The physical activity measure had to be scaled to fit the ABM. In the previous study with the same ABM, the mean value of physical activity was set at 1.53 [12]. Therefore, we computed a new variable named the physical activity level (PAL) by dividing the steps by 10,000 and multiplying by 1.53. The mean PAL value in our dataset was 1.50, with a minimum of 0.45 and a maximum of 4.27.

#### Family Affluence

A measurement of the influences of the social environment was needed as a second input parameter of the ABM. The Family Affluence Scale (FAS) was used as a measure of socioeconomic status [23]. The FAS is a self-reported measure of family affluence and is an effective tool for assessing socioeconomic status in adolescents [38]. The participants were asked sets of questions (eg, “How many cars does your family own?” and “How often do you go on a holiday outside of the Netherlands?”). All answers (range 0-13) were summed (mean_FAS_ 4.01, SD 1.52), reflected, and divided by the number of items (alpha=.41) to fit the model. This resulted in an environmental variable (*env*) with a value between 0 and 2 in which a higher *env* value reflects a lower family affluence.

#### Sociometric Nominations

In each wave, participants nominated peers from the same class by 6 sociometric questions based on the study by Starkey et al [24]. Participants received the questions at a random time during the day and nominated peers by clicking on their names in a list on the research smartphone. They were required to nominate at least one other peer, and no maximum on the peers nominated was given (note that self-nominations were not possible). For an overview of the questions, see Multimedia Appendix 1 [4,39,40].

#### Centrality

The social network characteristics at the individual level were calculated with the Python3 [41] package NetworkX 2.1 [42]. For an overview of the centrality measures, see Table 1. The individual’s betweenness centrality did not correlate with in-degree centrality or closeness centrality, but in-degree centrality did correlate with closeness centrality (*r*_457_=0.58; *P*<.001).

**Table 1:** Descriptive statistics for the individual- and group-level variables.

Variable name			Mean (SD)	Minimum	Maximum
**Individual characteristics (centrality; N=451)**					
	In-degree		12.27 (4.15)	4.00	27.00
	Betweenness		0.01 (0.02)	0.00	0.12
	Closeness		0.78 (0.11)	0.49	1.00
**Network characteristics (N=26)**					
	Density		0.72 (0.11)	0.46	0.90
	**Centralization**				
		In-degree	0.20 (0.08)	0.07	0.40
		Betweennes	0.04 (0.03)	0.01	0.09
		Closeness	0.22 (0.08)	0.09	0.39

#### Density and Centralization

Density and 3 centralization measures were calculated for each class. The density was calculated by taking the number of ties present in a social network and dividing this by the number of all possible ties, resulting in a number ranging from 0 (noncohesive network) to 1 (very cohesive network). In-degree centralization, betweenness centralization, and closeness centralization were calculated with the *igraph package* in the statistical computing package RStudio [43], resulting in a number ranging from 0 (*noncentralized network*) to 1 (*very centralized network*). The density and centralization scores were normalized given the different network sizes. For an overview of the density and centralization scores, see Table 1.

### Design

#### Social Networks

On the basis of the sociometric nominations, a directed social network was constructed for each classroom. A directional social network comprises nodes that represent the participants within a class and edges representing (weighted) connection between 2 nodes (referred to as *edge*).

The weight is defined as the sum of nominations of a participant toward another, divided by the total number of nomination questions. Because 2 participants could nominate each other, the edges in the network are directional (represented by the arrow of the edge). As participants nominated peers on multiple sociometric questions, each edge was associated with a *connection weight* ranging from 0 (zero nominations) to 1 (all 6 nominations). The more nominations a participant gave to another peer, the stronger the edge’s connection weight. Duplicate nominations were omitted (as a participant could nominate the same peers on the same items across waves), resulting in a maximum of 6 nominations toward another peer within all 4 waves.

#### Agent-Based Model

Computational models can be defined “as an abstract and simplified representation of a given reality, either already existing or just planned. Models are commonly defined to study and explain observed phenomena or to foresee future phenomena” [44].

ABMs are a particular category of computational models for simulating the communication among the agents in a common environment to understand their behavior. For this study, we relied on a previously validated ABM developed by Giabbanelli et al [21] and enriched by several adaptations [12,20].

Giabbanelli’s [21] model was used as it accounted for the interaction of social networks with environmental factors, unlike earlier related computational models for social network interventions. In this model, individuals influence each other with respect to physical activity that might change also depending on the agent’s physical environment. Their factor analysis on synthetic and real-world social networks showed that the environment was a crucial parameter for changes in bodyweight (their health behavior of interest). This particular model was favored as it was a good complement to the collected *MyMovez* data. Many previous studies used more complex models incorporating multiple parameters but based them on synthetic datasets. The purpose of this study was to use data collected from real human relations and behaviors, and this model was a good fit for the observed data.

The ABM simulates the spread of physical activity within social networks (classes), that is, simulating the spread of the intervention’s effect through the classes. We assumed that physical activity spreads throughout the relationships and depends on the physical environment. Each agent, in our case participants within a class, was assigned 2 input parameters before running the simulations—the PAL and the *env* parameter. Yearlong simulations were run for each of the social network intervention strategies and for each class. During each step (represented by a single day) of the simulation, a PAL value was derived for each agent based on the social influence and the environmental influence. The social influence comes from all the peers who are connected to the agent. It is based on the connection weights between agent’s peers and the associated peers’ PAL. The environmental influence is the effect of the agent’s family affluence, represented by *env*. The ABM does not make assumptions regarding probability of diffusion across ties.

Each simulation step potentially updates the agent’s PAL and was calculated in 3 phases, similarly as presented by Giabbanelli et al [21]. First, the social influence parameter was calculated, coming from the adolescent’s peers (dependent on peers’ PAL and connection weights). Second, the social influence with the agent’s environmental influence (ie, *env*) was combined in a single parameter, called the socioenvironmental influence. Third, the socioenvironmental influence parameter was compared with a predefined threshold to decide if agent’s PAL will be modified or remain the same.

See Multimedia Appendix 2 for a detailed description of the used ABMs. Multimedia Appendix 2 presents the ABM’s mathematical representation and gives more information about the model’s thresholds [45].

#### Interventions

A total of 5 conditions were created based on 4 social network intervention strategies and a control condition (no intervention). In the centrality-based intervention conditions (ie, in-degree, betweenness, and closeness centrality), the top 15% of participants with the highest centrality were assigned as influence agents. When participants above and below the cutoff score had the same centrality scores, random participants from these cases were assigned as influence agents. In the *random agent* intervention condition, 15% of influence agents were randomly selected out of all participants in a classroom. To diminish the possible effect of selecting a particular set of influence agents in the random agent condition, 100 interventions were simulated and averaged afterward to provide a single outcome value. In the *control condition*, no intervention was simulated.

All interventions were based on the assumption that the training sessions of the social network interventions were able to increase the physical activity of the influence agents at the start of the intervention. Therefore, all influence agents received an artificial increase of 17% in their initial PAL based on the outcomes of a previous behavioral intervention [12,46]. After the increase in PAL of the influence agents, the intervention simulations were run for 365 days (day 0-364). The effectiveness of the health interventions was expressed as the *success rate*, the percentage of increase in a class’s PAL from the start (day 0) to the end (day 364) of the simulation.

### Ethics Approval and Consent to Participate

Informed consent was obtained from 1 of the parents of the participants in the *MyMovez* project. Study procedures were approved by the Ethics Committee of the Radboud University (ECSW2014–100614-222).

## Results

### Simulating Physical Activity

**Figure 3:**
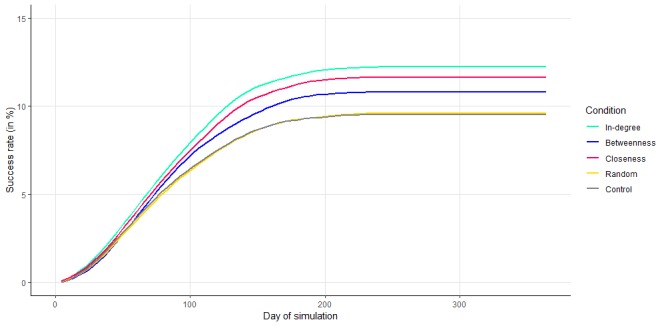
Intervention outcomes. Average success rate for the conditions over one-year simulation.

The simulations were used to observe the spread of physical activity among peers in the classes and determine the success rate of the different interventions. Figure 3 illustrates the trajectory of the averaged PAL of the classes for all the different simulated interventions for the 1-year period. What stands out from Figure 3 is that all simulated interventions increase the average physical activity of the networks. However, there are differences between the interventions in the increase of physical activity. A detailed overview of the interventions’ success rates for all conditions can be found in Multimedia Appendix 3.

As a first step, we tested the overall differences among all conditions. A linear mixed-effects model was run [47], with success rate as the dependent variable, condition as the predictor, and random intercepts per class. Mauchly test indicated that the assumption of sphericity was not met (*W*=0.00, *P*<.001, *ε*=.41). Therefore, all degrees of freedom were corrected by using the Huynh-Feldt estimation of sphericity. The repeated measures analysis of variance showed that the simulated interventions differed from one another (*F*_1.66,41.42_=7.72, *P*=.002, ε=.01). To investigate differences among the conditions as proposed in the hypotheses, planned contrasts (Helmert coding scheme) were used. In addition, all *P* values were corrected by using the Satterthwaite method as suggested by Luke [48].

### Selecting Influence Agents

For checking model validity, the first planned contrast was used to compare the 4 social network intervention conditions with the control condition (no intervention). The contrast revealed that the success rates of the social network interventions (11.28%) were higher than the control condition (9.76%; beta=.30; *t*_100_=3.30; *P*=.001). This means that the interventions were more successful in increasing physical activity than in the absence of interventions. Therefore, we presumed that ABM is a valid tool to simulate social network interventions.

To test the first hypothesis (H1), the second planned contrast compared the 3 centrality social network intervention conditions (ie, in-degree, betweenness, and closeness centrality conditions) with the random agent condition. The averaged success rate of the centrality social network intervention conditions (11.74%) was higher than the success rate in the random agent condition (9.90%; beta=.46; *t*_100_=3.86; *P*<.001). This means that having central influence agents is more effective in increasing physical activity than having randomly sampled individuals in a network.

To test the second hypothesis (H2), the third and fourth planned contrasts compared the differences within the 3 centrality social network intervention conditions. The third contrast compared the betweenness and closeness centrality conditions (11.57%) with the in-degree condition (12.08%). The success rates did not differ from each other (beta=−.17; *t*_100_=−1.00; *P*=.32). The fourth contrast compared the closeness centrality condition with the betweenness centrality condition. The success rates of the closeness centrality condition (12.16%) were higher than the betweenness centrality condition (10.98%; beta=.59; *t*_100_=2.02; *P*=.046). This means that we did not find evidence that the closeness centrality condition outperformed the in-degree centrality condition, but the betweenness centrality condition was less effective in increasing physical activity in the networks compared with the in-degree and the closeness centrality conditions.

### Network Characteristics

The success rates of the social network interventions varied among classes, as can be seen in Multimedia Appendix 3. A few classes stayed neutral to the interventions or even encountered negative effects (classes 101 and 129 showed PAL decrease), whereas other classes showed >30% average PAL increase over a year of simulation of particular intervention condition. Therefore, we investigated the effect of structural properties of the classes (ie, density, in-degree centralization, betweenness centralization, and closeness centralization) on the success rates of the interventions. More specifically, we added the different structural properties as moderators to the mixed-effects model. Table 2 displays the correlation coefficients of the 4 social network interventions and the 4 structural network properties. For a detailed overview of the structural properties per class, see Multimedia Appendix 4.

**Table 2:** Correlations between social network interventions and network structures.

Network structures	Network interventions			
	In-degree	Betweenness	Closeness	Random agent
Density	−0.37	−0.33	−0.35	−0.34
In-degree centralization	0.58^a^	0.57^a^	0.58^a^	0.56^a^
Betweenness centralization	0.26	0.26	0.26	0.21
Closeness centralization	0.35	0.30	0.33	0.30

^a^*P*<.05.

The third hypothesis (H3) predicted that the interventions would be more effective in classes with high density. To test whether the density of the class moderated the effectiveness of the different interventions, the same mixed-effects model was run with the addition of the interaction effect of density (standardized). The analysis showed that there was no significant direct effect of density on the success rate (beta=−3.17; *t*_24_=−1.58; *P*=.08). This means that the success rates were not higher in classes with high density than in classes with low density. In addition, no significant interaction effects of the planned contrasts of the social network conditions and the density of class were observed. This means that we did not find evidence to support the hypothesis (H3) that social network interventions were more effective in classes with high density compared with classes with low density.

The last 3 hypotheses (H4a, H4b and H4c) predicted that the interventions would be more effective in classes with high centralization based on the centrality measure that was used. For these analyses, the contrasts were changed per hypothesis, so that the centrality measure in focus was contrasted with the other social network interventions. For these 3 hypotheses, the same mixed-effects model was used, with the addition of the interaction effect of centralization.

**Figure 4:**
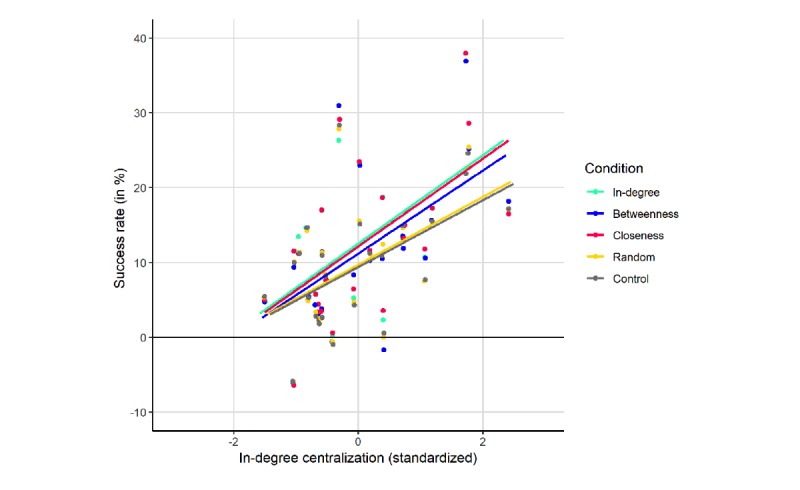
Effect of in-degree centralization on the success rates per condition.

The first linear mixed-effects model investigated in-degree centralization (H4a) and showed that there was a direct effect of in-degree centralization on the success rate (beta=5.27; *t*_9.94_=3.55; *P*=.002). As can be seen in Figure 4, the social network interventions were more effective in classes with high in-degree centralization. This means that social network interventions are more effective when the class is more centralized around some in-degree central individuals. In addition, we looked at the interaction of in-degree centralization and the planned contrast of the in-degree centrality condition versus the other social network interventions. This interaction effect was nonsignificant (beta=.15; *t*_39.76_=1.26; *P*=.21). This means the effect of in-degree centralization on the success rates was not stronger in the in-degree centrality condition than in the other social network conditions.

The second linear mixed-effects model investigated betweenness centralization (H4b) and showed that there was no direct effect of betweenness centralization on the success (beta=2.22; *t*_9.94_=1.25; *P*=.22). This means that the social network interventions were not more effective in classes with high betweenness centralization compared with classes with low betweenness centralization. In addition, the interaction effect was nonsignificant (beta=.09; *t*_39.76_=0.73; *P*=.45). This means that the effect of betweenness centralization on the success rates was not stronger in the betweenness centrality condition than in the other social network conditions.

The last linear mixed-effects model investigated closeness centralization (H4c) and showed that there was no direct effect of closeness centralization on the success rate (beta=2.88; *t*_9.94_=6.66; *P*=.11). This means that the interventions were not more effective in classes with high closeness centralization compared with low closeness centralization. In addition, the interaction effect was nonsignificant (beta=.11; *t*_39.76_=0.93; *P*=.36). This means that the effect of closeness centralization on the success rates was not stronger in the closeness centrality condition than in the other social network conditions.

Given these results, our hypotheses that the effectiveness of the simulated social network interventions would be greater in classes with high centralization than classes with low centralization were rejected. We only found evidence that social network interventions were more effective in high in-degree centralized classrooms, irrespective of the type of social network intervention used.

## Discussion

### Principal Findings

The aim of this study was to test which selection criterion to determine influence agents in a social network intervention resulted in the biggest increase in physical activity in the social network. To test different selection criteria for influence agents, an ABM was used to simulate different selection criteria for social network interventions and observe the intervention’s effect on the average physical activity of the classroom. In addition, the study investigated whether social network interventions were more effective in some classes than others based on their particular network characteristics.

The general effectiveness of social network interventions was compared with the control condition. The results showed that the increase in physical activity was of greater magnitude in social network interventions than in the control condition. This demonstrates that an increase in physical activity of a small group of individuals has the potential to spread to peers in the social network. Therefore, the ABM produced results in line with the social network theory, which predicts that behaviors spread in social networks [1,2,3]. We, therefore, assumed that our model was a valid tool to test our hypotheses.

In addition, the effect was stronger for the centrality-based social network intervention conditions compared with the random influence agent condition. This is not in line with the results of the first model of El-Sayed et al [13] who concluded (also based on simulations of literature-based parameters) that well-connected influence agents had little or no added value compared with random influence agents. This difference may be a result of the different model specifications in the 2 studies. In addition, the outcome variable in the study by El-Sayed et al [13] was the prevalence of obesity. On the contrary, the results of this study are in line with the second set of simulations of artificially high parameter models of El-Sayed et al [13] and Zhang et al [10]. These results corroborate the idea that central individuals hold an important position within their social networks [25]. Taking a random subsample of the participants as influence agents is not as effective as strategically located influence agents. Therefore, researchers should carefully select influence agents based on their position in the social network, as suggested by Borgatti [26] and Valente and Pumpuang [30]. When researchers are unable to strategically select the influence agents, Bahr et al recommend increasing the percentage of random influence agents to obtain the same success rates as the centrality conditions with 15% of the class as influence agents [49].

Contrary to expectations, no difference was observed between the in-degree centrality condition and the closeness centrality condition, as suggested by Borgatti [26] and Valente [30]. An explanation could be that Valente’s argument [27] that in-degree agents are most often the popular individuals and not willing to change their behavior does not hold for simulated intervention. In the simulations, the artificial increase of physical activity of the influence agents was the same for the in-degree centrality condition and the closeness centrality condition. In contrast, a difference between the closeness centrality condition and the betweenness centrality condition was observed. In accordance with Borgatti’s [28] reasoning that positive behavior should be promoted via closeness central agents, we observed that the closeness centrality condition had a higher success rate than the betweenness centrality condition. This corroborates the idea that when researchers want to increase a positive behavior, closeness centrality influence agents should be selected.

Finally, this study looked at the moderating role of structural characteristics of the class on the effectiveness of the social network interventions. The results showed that the density of the class did not affect the success rates of the social network interventions. This is not in line with social network theory, which argues that innovations spread quicker through highly connected networks [32]. We also anticipated that the specific centrality conditions were most effective as the classes were more centralized on the relevant centrality measure. However, the results indicated that only in-degree centralization had a direct effect on the success rates. This means that social network interventions are more effective when classes have a small number of individuals who receive the most nominations. The subsequent analyses showed that this effect was not stronger in the in-degree centrality condition than in the other social network intervention conditions. Therefore, we can conclude that social network interventions work well in classes with high in-degree centralization irrespective of the selection criterion used.

This study advanced the field of social network interventions and the use of ABMs for better understanding interventions in numerous ways. This study was one of the first to use simulations to test the difference among the selection criteria for the influence agents in social network health interventions. In addition, this study used empirical data as input for the model. The next step in the interplay between health interventions and computational models will be to replicate these simulated results with empirical data of social network health interventions.

The study provides implications for future research and can advise social network researchers. First, this study supports the idea that social network interventions can be an effective strategy to increase physical activity in the classroom. Second, it stresses the importance of strategically selecting the most central individuals as influence agents. Finally, the composition of the class can influence the effectiveness of social network interventions. In addition, this study shows the applicability of simulations to help researchers design the most effective interventions.

### Comparison With Previous Studies

ABMs have been used previously to study the spread of health behaviors in simulated social environments after hypothetical interventions. For example, an ABM was used to investigate the spread of obesity in artificial participants after multiple obesity prevention campaigns [13]. The use of ABMs to investigate the spread of obesity was further refined by using the body mass index of an observed sample of participants and the addition of a socioenvironmental factor [21]. However, no behavioral data were available, so physical activity was imputed based on a random distribution. A subsequent study improved the previously mentioned ABM by incorporating individual thresholds for the change in health behaviors [12]. Our previous study used this model, but here we applied it to observed behavioral and sociometric data [20]. The previously mentioned ABMs [12,20,21] showed the same results as this study in that the simulations of interventions showed an increase that attenuated over time.

On the basis of different ABMs, 2 other studies have used agent-based simulations to investigate the effectiveness of different types of influence agents in social network interventions [9,10] but both with a slightly different aim. The study by Zhang et al [10] examined only the difference between randomly selected and in-degree central influence agents. Their conclusion aligns with the findings from this study in that physical activity increases more in the intervention that uses influence agents based on centrality compared with the intervention that uses random influence agents.

The study by Badham et al [9] matches the research question of this study more closely, that is, the study looked at the 3 different types of centrality measures. However, the outcome of the simulations was the amount of time (number of iterations) before the entire network adopted a behavior. In other words, the study by Badham et al [9] focused on the speed of adoption of the intervention and not on the magnitude of the behavior change after the simulated interventions. Despite the different outcome variable, the studies showed comparable outcomes to the findings of this study. More specifically, the most effective interventions are those with influence agents based on centrality (ie, in-degree, betweenness, and closeness centrality). Although the study by Badham et al [9] did not formally test the differences among the centrality measures, the observed steps to saturation did not indicate that there was a difference among them.

### Limitations

To interpret the results of the simulation of social network interventions, a number of limitations have to be discussed. First, this study was based on the assumption that researchers were able to increase the amount of physical activity of the influence agents. However, it could be that this does not reflect the field experiments that train influence agents to become more active. In addition, increasing the targeted health behavior is only part of the influence agents’ training. For example, most training sessions in social network interventions also focus on how the influence agent could communicate the health message in an informal way. This type of health promotion was not a part of the ABM that we used. Future studies could also imitate other aspects of a successful training. For example, researchers could consider increasing the number or the weight of the connections to reflect the communication component of the influence agents’ training. Along the same lines, the success rates of the intervention are based on the embedded assumptions in the model of how people influence each other. In our model, the assumption was that the increase in physical activity diffuses over time. However, adopting a contagion framework, which looks at how many peers should increase in physical activity before the individual’s physical activity increases, might lead to different success rates of the interventions.

Second, the employed ABM comes with a set of limitations. For example, based on the mathematical characteristics of the model, the ABM’s outcome has an initial increase and reaches an equilibrium state after a particular time in the simulations, as shown in Figure 3. Consequently, the control condition also increased in physical activity, contrary to the usually observed decrease among the youth [50]. Therefore, caution is warranted in interpreting the absolute increase in classes’ physical activity. Rather, we want to emphasize that the results focused on the relative differences among the selection criteria. In addition, the ABM outcomes enabled us to discuss the effects of *simulated* health interventions. Although the ABM has been validated and tuned to the empirical data, the presented simulation effects should be interpreted with caution. Following this limitation, in our next study we intend to perform similar statistical analyses on the empirical data when the intervention outcomes of the *MyMovez* project are available.

Third, the applied analyses were all based on data aggregated on a classroom level. However, we realize the importance of conducting more elaborate individual-level analyses by including personal characteristics, such as sex, personality traits, individual physical activity, or role in the social network. These personal characteristics can moderate the effect of the health intervention. By including more personal information, the ABM can be better specified. Adopting personality traits could help us understand how an individual perceives and reacts to peer behaviors as well as learn about individuals’ contributions to the class behavior.

### Conclusions

In conclusion, we demonstrated the advantages of applying social network analyses and simulations to understanding social networks’ characteristics and performing detailed simulations on peer influences. We advise future researchers to perform such simulations on peer influences, whenever possible, *before* doing real-world interventions to maximize the success rate of their interventions. This information can help in designing more effective social network health interventions.

## Appendix

Multimedia Appendix 1 Peer nomination questions.

Multimedia Appendix 2 Model Description.

Multimedia Appendix 3 Success rates per class of one year simulations of the interventions (in percentages).

Multimedia Appendix 4 Structural network parameters per class based on the weighted ties.

## Abbreviations

ABM: agent-based model
FAS: Family Affluence Scale
PAL: physical activity level
