# Supplementary material for: Identifying Influence Agents That Promote Physical Activity Through the Simulation of Social Network Interventions: Agent-Based Modeling Study
Source: J Med Internet Res. 2019 Aug 5;21(8):e12914. doi: 10.2196/12914 (PMC6699133; doi:10.2196/12914)
Supplement: Multimedia Appendix 1 [file jmir_v21i8e12914_app1.pdf]

Appendix A

Peer nomination questions

Measure Description	Literature Reference	Survey
Advice network	Campbell et al., 2008 [4]	1 item assessing to who participants go for advice
Friends network	Brechwald & Prinstein, 2011 [48]	1 item assessing to who participants are friends
Innovators in the network	not based on reference	1 item assessing who most often have the newest products & clothes
Leader network	Campbell et al., 2008 [4]	1 item assessing who participants consider as leaders
Respect network	Campbell et al., 2008 [4]	1 item assessing who participants respect
Social facilitation network	Salvy et al., 2012 [49]	1 item assessing who participants hang out / have contact with
Want to be network	Campbell et al., 2008 [4]	1 item assessing who participants want to be like
