# Supplementary material for: Identifying Influence Agents That Promote Physical Activity Through the Simulation of Social Network Interventions: Agent-Based Modeling Study
Source: J Med Internet Res. 2019 Aug 5;21(8):e12914. doi: 10.2196/12914 (PMC6699133; doi:10.2196/12914)
Supplement: Multimedia Appendix 2 [file jmir_v21i8e12914_app2.pdf]

Appendix B

The model implementation can be generalized in three steps. In the first step, we calculated the social network peer influence, combining the PAL and the *env* parameter of an agent. The combined PAL social influence was calculated as following:

$$inf_{PAL_i}(t) = \frac{\sum_j \left(PAL_j(t-1) - PAL_i(t-1)\right) \times \omega_{(i,j)}}{\sum_j \omega_{(i,j)}}$$

The PAL influence is the sum of differences between each peer's PAL and the agent's PAL multiplied by the weight of the agents' connections, calculated as explained in Section 2.3.2. In cases when $inf_{PAL_i}(t) > 0$, the peer influence on agent I's PAL was positive and might contribute to increasing an agent's PAL. The *env* value was constant during the simulation and was considered beneficial when 0 < *env* < 1 and harmful when 1 < *env* < 2. Previous studies have shown that children with lower family affluence were less likely to be physically active than children with higher affluence [50]. Therefore, beneficial *env* values will further increase the PAL, while harmful *env* values will reduce the PAL [11]. In the second step, the two parameters from above were combined in order to get a socio-environmental influence parameter:

$$inf_{PAL_i}(t), env = env \times inf_{PAL_i}(t), \qquad if \;\; inf_{PAL_i}(t) < 0$$

$$inf_{PAL_i}(t), env = \frac{inf_{PAL_i}(t)}{env}, \qquad if \;\; inf_{PAL_i}(t) \geq 0$$

In the last step, the socio-environmental parameter was compared with a given threshold to determine whether the amount of the socio-environmental influence was enough to cause a behavior change. Therefore, equations 4 and 5 show the value for an agent's PAL in the next time step *t*, where *factor* = 1 + I_{PAL}, in case $inf_{PAL_i}(t), env > 0$,

and factor = 1 - I_{PAL}, otherwise.

$$PAL_i(t) = PAL_i(t-1), \qquad if \;\; \left|inf_{PAL_i}(t), env\right| < T_{PAL}$$

$$PAL_i(t) = PAL_i(t-1) \times factor, \qquad if \ \left|inf_{PAL_i}(t), env\right| \geq T_{PAL}$$

Please refer to our previous work [9] for a more detailed description of the model and the procedures that were used for the validation of the model and the parameter tuning. Slight model modifications are applied in this paper, as we are interested in simulating nomination networks (influence networks are used in the previous work). The direction of spreading the behavior is from node *i* to node *j* in contrast to influence models where behavior is spread from node *j* to node *i*.
