# Supplementary material for: Identifying Influence Agents That Promote Physical Activity Through the Simulation of Social Network Interventions: Agent-Based Modeling Study
Source: J Med Internet Res. 2019 Aug 5;21(8):e12914. doi: 10.2196/12914 (PMC6699133; doi:10.2196/12914)
Supplement: Multimedia Appendix 3 [file jmir_v21i8e12914_app3.pdf]

Appendix C

Success rates per class of one year simulations of the interventions (in percentages)

Class ID	In-degree centrality	Betweenness centrality	Closeness centrality	Random agent	Control
67	10.70	10.60	11.80	7.50	7.69
71	13.22	13.51	13.22	14.66	14.93
72	26.31	30.95	29.12	27.85	28.35
74	2.34	-1.69	3.56	0.00	0.58
77	5.24	8.32	6.44	4.70	4.27
78	11.59	10.26	11.59	11.12	11.24
79	5.50	5.42	5.01	4.86	5.29
81	14.66	14.62	14.66	14.23	14.69
83	7.62	7.62	7.62	8.43	8.16
86	4.72	4.74	5.03	5.35	5.46
100	4.40	3.19	4.40	2.54	1.81
101	0.27	-0.62	0.61	-0.55	-0.96
103	23.31	22.98	23.45	15.56	15.10
121	37.95	36.91	37.95	22.61	21.99
122	3.36	3.36	3.36	2.35	2.27
125	17.01	11.42	17.01	11.37	10.94
126	5.83	4.33	5.74	3.36	2.80
127	3.48	3.83	3.48	2.49	2.66
129	-6.08	-6.08	-6.41	-5.88	-5.88

130	17.26	15.67	17.26	15.37	15.54
131	18.18	18.18	16.51	17.12	17.16
133	18.73	10.49	18.65	12.41	10.89
135	13.42	11.15	11.15	11.40	11.17
136	11.51	9.33	11.51	10.08	9.97
138	28.60	25.20	28.60	25.44	24.59
139	14.94	11.87	14.94	13.01	13.15
