# Supplementary material for: Identifying Influence Agents That Promote Physical Activity Through the Simulation of Social Network Interventions: Agent-Based Modeling Study
Source: J Med Internet Res. 2019 Aug 5;21(8):e12914. doi: 10.2196/12914 (PMC6699133; doi:10.2196/12914)
Supplement: Multimedia Appendix 4 [file jmir_v21i8e12914_app4.pdf]

Appendix D

Structural network parameters per class based on the weighted ties.

Class ID	Number of participants	Sex Ratio	Number of Edges	Density	In-degree Centralization	Betweenness Centralization	Closeness Centralization
67	18	M:50, F:50	205	0.67	0.29	0.05	0.25
71	20	M:65, F:35	247	0.65	0.26	0.03	0.25
72	20	M:70, F:30	238	0.63	0.17	0.06	0.27
74	12	M:50, F:50	104	0.79	0.23	0.03	0.19
77	19	M:31, F:68	223	0.65	0.19	0.05	0.26
78	20	M:50, F:50	303	0.80	0.21	0.02	0.16
79	25	M:48, F:52	275	0.46	0.13	0.05	0.39
81	28	M:53, F:46	663	0.88	0.13	0.01	0.10
83	14	M:50, F:50	142	0.78	0.15	0.04	0.17
86	16	M:50, F:50	192	0.80	0.07	0.05	0.15
100	20	M:50, F:50	288	0.76	0.14	0.02	0.22
101	18	M:38, F:61	205	0.67	0.16	0.07	0.24

103	17	M:70, F:29	221	0.81	0.20	0.01	0.15
121	14	M:71, F:28	110	0.60	0.34	0.09	0.30
122	11	M:81, F:18	84	0.76	0.14	0.02	0.22
125	17	M:41, F:58	200	0.74	0.15	0.01	0.26
126	11	M:63, F:36	96	0.87	0.14	0.02	0.11
127	14	M:64, F:35	129	0.71	0.15	0.05	0.25
129	9	M:66, F:33	65	0.90	0.11	0.02	0.09
130	21	M:47, F:52	259	0.62	0.30	0.08	0.27
131	11	M:72, F:27	70	0.64	0.40	0.08	0.30
133	20	M:40, F:60	217	0.57	0.23	0.07	0.31
135	18	M:44, F:55	272	0.89	0.12	0.01	0.09
136	19	M:47, F:52	268	0.78	0.11	0.02	0.16
138	20	M:35, F:65	255	0.67	0.35	0.03	0.25
139	19	M:31, F:68	201	0.59	0.26	0.09	0.29
